# Peptide Gelators to Template Inorganic Nanoparticle Formation

**DOI:** 10.3390/gels7010014

**Published:** 2021-02-02

**Authors:** Ottavia Bellotto, Maria C. Cringoli, Siglinda Perathoner, Paolo Fornasiero, Silvia Marchesan

**Affiliations:** 1 Chemical and Pharmaceutical Sciences Department, University of Trieste, 34127 Trieste, Italy; ottavia.bellotto@phd.units.it (O.B.); mcringoli@units.it (M.C.C.); pfornasiero@units.it (P.F.); 2 INSTM, Unit of Trieste, 34127 Trieste, Italy; 3 Dipartimento di Scienze Chimiche, Biologiche, Farmaceutiche e Ambientali, University of Messina, 98168 Messina, Italy; siglinda.perathon@unime.it; 4 INSTM, Unit of Messina, 98168 Messina, Italy; 5 Istituto di Chimica dei Composti Organometallici, Consiglio Nazionale delle Ricerche (ICCOM-CNR), 34127 Trieste, Italy

**Keywords:** peptides, gels, inorganic nanoparticles, nanowires, nanotubes, nanostructures, metal, metal oxides, self-assembly

## Abstract

The use of peptides to template inorganic nanoparticle formation has attracted great interest as a green route to advance structures with innovative physicochemical properties for a variety of applications that range from biomedicine and sensing, to catalysis. In particular, short-peptide gelators offer the advantage of providing dynamic supramolecular environments for the templating effect on the formation of inorganic nanoparticles directly in the resulting gels, and ideally without using further reductants or chemical reagents. This mini-review describes the recent progress in the field to outline future research directions towards dynamic functional materials that exploit the synergy between supramolecular chemistry, nanoscience, and the interface between organic and inorganic components for advanced performance.

## 1. Introduction

Supramolecular gels composed of peptides have attracted great interest in recent years across multidisciplinary research communities for a variety of reasons [1]. Firstly, peptides, especially short ones, can readily be prepared by well-established solid-phase methods, also for ease of purification [2]. Secondly, they allow greater chemical diversity, relative to other classes of compounds, which has been further expanded by the introduction of a large variety of non-natural amino acids [3,4]. Thirdly, as the complex function of proteins can be pinned to specific sets of peptide sequences, it has been shown that peptides can encode for at least some of protein functionalities [5]. Fourth, when a function is encoded within a supramolecular system, it is possible to envisage its on/off switching by means of assembly/disassembly cycles, a convenient feature especially for biomedical applications [6]. Finally, peptides are inherently biodegradable and biocompatible, thus facilitating the use of green solvents, such as water, for their final systems that will not excessively persist in the environment; this is particularly relevant for the development of alternatives to traditional materials used in electronics [7].

**Figure 1:**
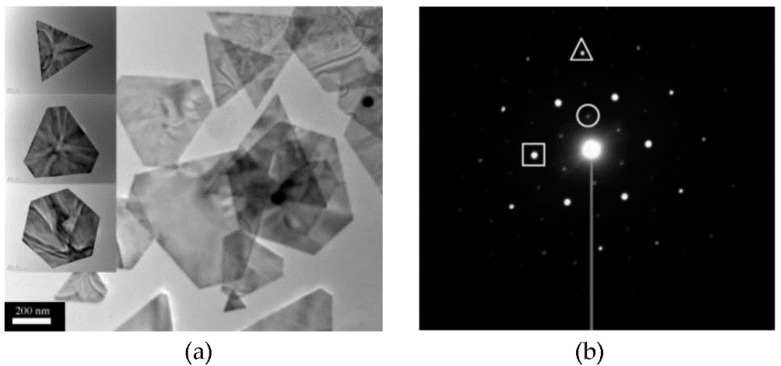
(**a**) TEM images of gold nanoplates obtained from the reduction of chloroaurate using seaweed extract. (**b**) The selective area electron diffraction (SAED) pattern of a triangular nanoplate. The strongest spots (square) could be assigned to the {220} reflections, the outer spots (triangle) with weaker intensity could be indexed to the {422} reflections, and the inner spots (circle) with the weakest intensity correspond to the formally forbidden 1/3 {422} reflections. Adapted with permission from [32]. Copyright © 2021, American Chemical Society.

However, peptide gels have also their limitations, and for this reason research has been very active on the development of composite or hybrid gels with additional chemical components to enhance their properties [8,9]. Examples show a wide range of chemical diversity, which includes synthetic polymers [10,11], polysaccharides [12] and nucleic acids [13], carbon nanostructures [14], polyoxometallates [15], metal-organic cages [16], and more. A particular class of additional components that deserves a separate discussion is inorganic nanoparticles (NPs). Several reviews have recently appeared on how to guide their formation using ionizing radiation [17], hydrothermal routes [18], microemulsions [19], microfluidics [20], aerogels [21], air/liquid interfaces [22], polymers [23], cyclodextrins [24] and host-guest interactions [25], ligands [26], and proteins [27]. In particular, it is well-known that inorganic nanoparticles do exist in nature [28], and their biosynthesis has been a source of inspiration for chemists for a long time [29] to develop green routes with fine control over their shape and size (Figure 1). This is particularly relevant for applications, for instance to achieve optimal performance of nanostructured catalysts [30], for the transition towards clean energy production and storage [31].

**Figure 2:**
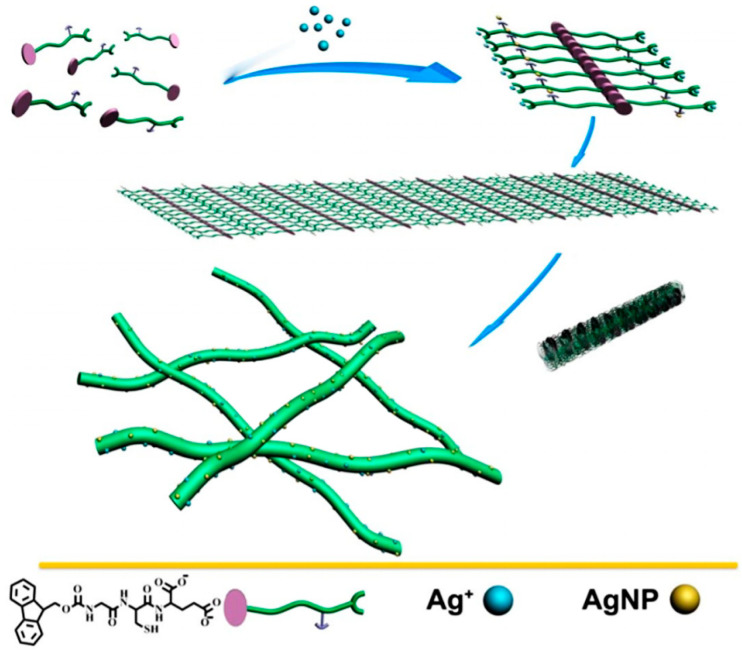
The templating effect of a tripeptide gelator bearing carboxylates and a thiol moiety for Ag^+^ coordination results in the formation of silver nanoparticles (AgNP) onto the gelling nanofibers that arise from the peptide stacks. Reprinted with permission from [46]. Copyright © 2021, American Chemical Society.

The process of biomineralization starts when the mineral inorganic precursors and specific organic biomolecules come into contact; often, the biomolecules that come into play are proteins [33]. However, there is large diversity (and lack of homology) in the amino acid sequence of the proteins involved in the process, when they are known [33]. Peptide sequences capable of templating inorganic NP formation typically display N-/O-/S-donor groups for metal coordination (Figure 2), although the relationship between peptide sequence and the templating effect has not yet been identified [34].

The general understanding is that peptides can bind to nuclei or metal nanoclusters and create a reducing environment that promotes metal ion reductions to metal NPs [35]. Among the various functional groups involved in the process, hydroxyl groups (e.g., found in Tyr, Ser, or DOPA [36] amino acids) have recently gained attention, as they are often found in other biomolecules, i.e., sugars and polyphenols, which act as reducing agents in the biosynthesis of metal nanoparticles [37]. Amines (e.g., from Lys) often exert a role as capping agents, as one of the steps in a mechanism whose complexity has not yet been completely clarified [38]. Cys-thiols are widely exploited for the coordination of gold (and silver) to favor the initial steps of nucleation. However, it should be kept in mind that a variety of factors and processes clearly come into play to define the resulting nanomaterial, including thermodynamic aspects, such as the rate of nucleation relative to that of crystal growth, and phenomena that include Ostwald ripening and stacking faults [39].

In other strategies, photoreduction is used, and several examples have been described for the in situ formation for instance of silver NPs [40,41,42]. Additionally, the photoreduction of gold can be performed on the surface of peptides as capping agents to stabilize the resulting NPs, for application in the biochemical field, such as siRNA vectors for oncotherapy [43]. Either strategy is particularly attractive as it permits to avoid the use of polluting chemical reagents or harsh reaction conditions.

Therefore, the combination of peptide gelling ability with their templating effects for inorganic NP formation is particularly attractive as a green and easy way to achieve composite or hybrid organic-inorganic nanostructured materials [44]. Furthermore, peptide gels can also provide colloidal stability to the NPs, as well as a convenient method for their physical embedment in space, and their controlled delivery over time [44]. There are already quite a few reports of peptide gels used to template inorganic NP formation as discussed in detail further below. This review will not focus on systems whereby NP formation is chemically driven by the addition of reductants or other reagents besides the gelator, nor on peptide gelation in the presence of pre-formed NPs, a topic that has already been reviewed elsewhere [45].

## 2. Inorganic Nanostructure-Templating Effects by Peptides

The conjugation of peptides and metallic NPs, especially gold and magnetic iron oxide NPs, is of particular interest in nanomedicine for the targeting of sub-cellular biological structures, such as proteins or genes, as has been already reviewed [47]. Silica nanostructures are also promising especially in light of their chemically rather inert nature, inability to conduct electrons and highly transparent nature, and the process of biomineralization has been a source of inspiration to use proteins to direct silica NP formation [48]. Elongated nanostructures have also attracted attention thanks to their inherent chemical, magnetic, electrical, and optical anisotropy that find application especially in sensing [49], but also in drug delivery [50]. Similarly, the ordered arrangement in space at the nanoscale of metal NPs, such as silver NPs, can be advantageous to control their plasmonic properties for surface enhanced Raman spectroscopy (SERS) [51].

Peptides that bind on NP surfaces are interesting because they can also be used for biological targeting and may play a role in determining NP biodegradation [52]. Early studies identified over 30 peptide sequences able to bind cobalt ions, and nearly 20 that bound silver ions by using phage-display and PCR methods [53]. The ions were reduced in situ into spherical or polyhedral-shaped nanoparticles of average diameters ranging from ca. 50 to 100 nm, but no clear rationale was found that could relate the amino acid sequence with nanoparticle formation, other than the presence of recurrent amino acids, such as cysteine (Cys), histidine (His), glutamic acid (Glu), isoleucine (Ile), tryptophan (Trp), and tyrosine (Tyr) [53]. However, no gel was formed.

In particular, Tyr redox activity on gold ions was already well known, as it was noted that alkylated-Tyr served as phase transfer, reducing and capping agent for the formation of gold-NPs at liquid-liquid or liquid-air interfaces [54]. Cys was also a well-known ligand for silver and gold ions. Several Cys-bearing peptide sequences have been described for their ability to promote gold nanocluster formation [55], as well as gold NPs as described further below. Additionally, the dimerized form of cysteine has been used to promote selenium (Se) NP formation, exploiting sulfur affinity for Se, although ascorbic acid as reducing agent was also needed [56]. Furthermore, the use of peptide ligands can be advantageous to induce a chiral templating effect; to this end, the tripeptide Tyr-Tyr-Cys was recently used as a ligand to promote the formation of chiral cobalt oxide NPs, and the use of N- or C-terminally protected analogs demonstrated an important role played also by the peptide carboxylate in the interactions with the metal [57]. Analogously, Trp-bearing dipeptide amphiphiles were used for the in situ formation of gold NPs, whereby different morphologies could be attained based on the specific porosity and the templating effect of the different hydrogelators [58].

As another strategy, also simple hydrophobic tripeptide gelators were shown to be prone to biomineralization, through the deposition in situ of luminescent cadmium sulfide (CdS) NPs, which demonstrated altered optoelectronic properties with a blue-shift, once they were anchored onto the fibrillary network of the resulting hydrogel [59]. However, it should be noted that in this case the peptide fibers did not catalyze NP formation, which was performed by using cadmium nitrate, sodium sulfide and cysteine [59]. Table 1 reports key examples of peptide gelators that were used to promote the in situ formation of inorganic NPs, and further below specific examples are described based on the nanostructure morphology attained and the metal used, which is relevant for the envisaged applications as discussed in each section.

### 2.1. Nanoparticles

#### 2.1.1. Gold (Au)

Gold nanoparticles are well-known for their physicochemical properties that find applications in a variety of fields. Gold NPs have been widely researched for their use in catalysis and beyond [71]. Gold is notable for its good biocompatibility and its surface plasmon oscillations are advantageous for labelling, imaging, and sensing [72]. Obviously, these properties find interesting applications in the biomedical field, both in the diagnosis and therapy of cancer [73], as well as in their combination in the modern field of theranostics [74]. It is, thus, clear that their combination with (bioactive) peptides is particularly attractive to develop biomaterials [75].

**Figure 3:**
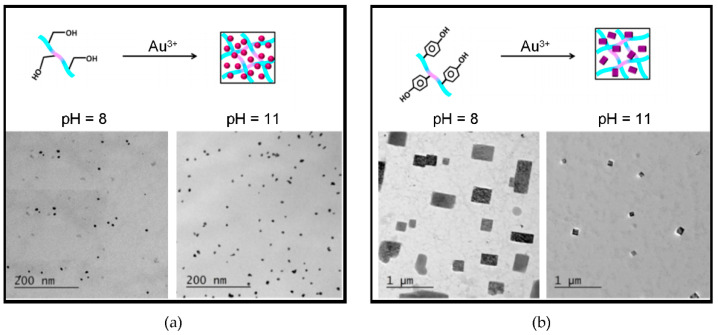
Self-assembling Fmoc-dipeptides template gold NPs with control over shape and size. (**a**) Fmoc-Phe-Ser yields spherical NPs. (**b**) Fmoc-Phe-Tyr yields rectangular plates. Adapted with permission from [65]. Copyright © 2021, American Chemical Society.

A series of peptide amphiphile gelators based on *N,N,N*-trimethylammonium Phe successfully templated gold NPs in various shapes and sizes, with preference over octahedral geometry upon optimization of experimental conditions [66]. Variations including Trp as reducing agent for Au^3+^ were also effective, and allowed to control the gold nanomorphology [58]. Other studies on dipeptide amphiphiles demonstrated that Trp was actually not necessary for Au NP formation; also aromatic N-methylation on Trp-bearing amphiphiles or amphiphiles based on Phe instead of Trp, allowed for the templating of spherical Au NPs with good size-control on the surface of nanofibers, showing that hydrophobic and confinement effects were more important [64]. Interestingly, NP-enriched hydrogels could be formed, and subsequent addition of toluene then led to a phase-transfer of both the gelator and gold NPs to the organic solvent to yield organogels [64]. Recently, Fmoc-dipeptide gelators were used to achieve morphological control over gold NP growth (Figure 3); in particular, Fmoc-Phe-Tyr acted as strong reductant, leading to rectangularly shaped gold NPs, while Fmoc-Phe-Ser acted as mild reductant leading to spherical gold NPs [65].

#### 2.1.2. Silver (Ag)

Silver NPs have been long-known for their good biocompatibility and antimicrobial activity, which find applications in the biomedical field [76], often combined with gels [77]. However, silver NPs are also attractive as conducting inks for use in electronics [78], and recently they are being re-discovered also as catalysts in a wide variety of organic transformations for the synthesis of fine chemicals [79]. Clearly, the development of green routes for their synthesis has become a key area of investigation [80].

The same gelators described above for the production of gold NPs [58] failed to produce silver NPs because of the undesired precipitation of AgCl due to the presence of chloride as the gelator counterion. A simple substitution towards aromatic carboxylate as counterions improved, at once, both the gelling ability of the peptide amphiphiles as well as their ability to template silver NPs, albeit with irregular shape [62].

In a different approach, silver photoreduction has been widely applied to obtain NPs templated by peptide gelators. For instance, a dipeptide with COOH groups as metal ligands was shown to promote sunlight-driven photoreduction of silver to form ultrasmall inorganic nanoparticles (1–3 nm diameter) embedded in a DMSO/water gel for envisaged applications as antimicrobial material [40]. Additionally a hexapeptide was used for the in situ UV-driven formation of 10–20 nm silver NPs, and the composite material showed antibacterial activity and good biocompatibility in vitro, as it was envisaged for wound healing applications [41]. Silver photoreduction was also successfully achieved for a bile acid-dipeptide conjugate; interestingly, the heterochiral dipeptide was effective for the purpose, while in the case of its diastereoisomer, photoreduction was not productive to form the silver NPs with antimicrobial activity [42].

Different was the case of a simple Fmoc-tripeptide with a Cys and Glu residues to provide coordinating ligands for Ag^+^ that gelled only in the presence of silver ions (shown in Figure 1), and at the same time, the assembling process templated silver NP formation on the peptide nanofibers [46]. The role of Ag^+^ in the gelation was confirmed by the addition of competing ligands, such as melamine or pyridine, which disrupted the gel that reformed upon addition of silver nitrate [46]. The hydrogel with silver NPs was effectively used for the degradation of methyl orange dye, and it also demonstrated antibacterial activity [46].

**Figure 4:**
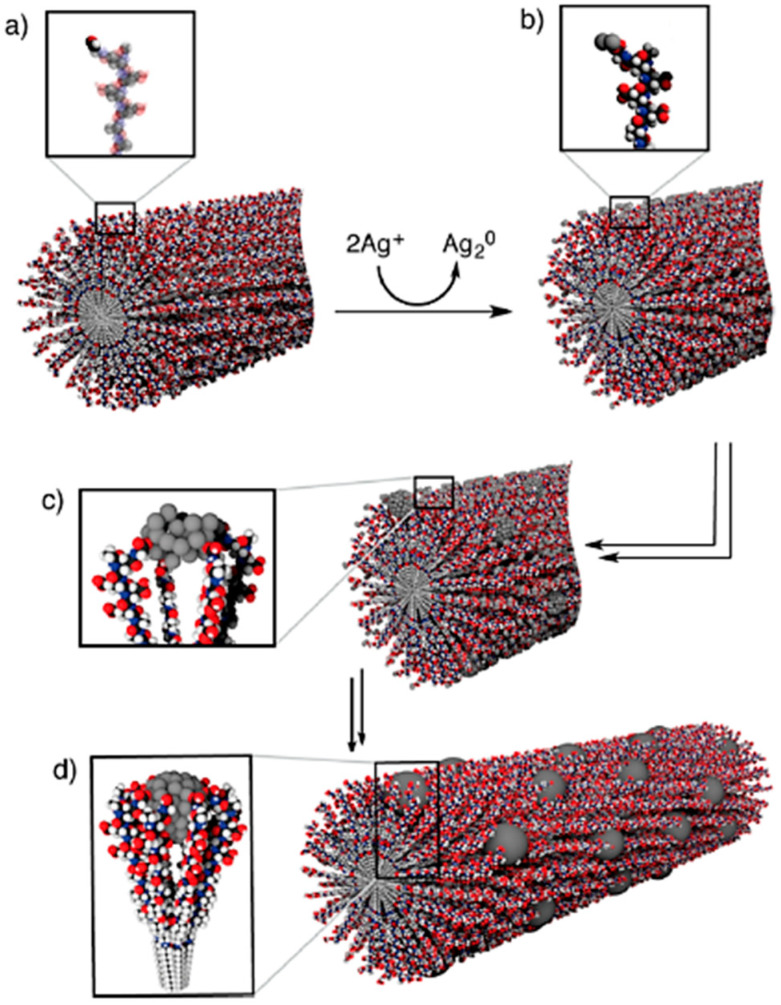
(**a**) Peptide amphiphile nanofiber with aldehyde groups on the surface. (**b**) Formation of silver clusters onto the nanofiber surface upon addition of the Tollen’s reagent. (**c**) Silver nanoclusters are stabilized by the Glu carboxylic cid residues. (**d**) Silver nanoclusters fuse into Ag NPs anchored onto the peptide amphiphile’s nanofiber surface. Reprinted with permission from [81]. Copyright © 2021, American Chemical Society.

Stupp and co-workers also showed the reduction of silver using the Tollen’s solution in contact with a peptide amphiphile hydrogelator, which led to the deposition of silver NPs onto the nanofibers (Figure 4), yielding hydrogels with antibacterial properties [81].

Furthermore, Cys(S-Bzl) thioether moieties were shown to direct the formation of silver NPs with spherical morphology and a diameter of 9 nm, while analogous peptides without the Cys moieties led to larger NPs due to the lack of control over NP formation, which was driven mainly by the peptide hydrophobic environment [61]. This study confirmed once more the importance of including suitable metal ligands in the peptide sequence to attain fine control over the size and morphology of the resulting NPs.

#### 2.1.3. Cadmium Sulfide (CdS)

Cadmium and, in particular, cadmium sulfide (CdS) have emerged as interesting nanomaterials for use in electronics [82]. In more recent applications, CdS NPs have also been combined with other metals, such as gold, for advanced performance in sensing, specifically for the detection of Cu^2+^ ions [83]. Stupp and co-workers developed peptide amphiphiles that could self-assemble into gelling fibers, whereby phosphoserine or the acidic groups effectively bound Cd^2+^ thus leading to the formation, upon addition of hyrogen disulfide, of 3–5 nm-wide CdS NPs onto the fibers’ surface [67].

#### 2.1.4. Platinum (Pt)

Pt NPs have been widely exploited by industry for their catalytic applications, such as their use in automotive converters as well as in petrochemical cracking [84], and supported platinum is also very useful in electrocatalysis for fuel cells and CO_2_ conversion [85]. More recently, Pt NPs also gained attention in the biomedical field, and for a range of uses that are based on their antimicrobial, antioxidant, and anticancer properties [86]. Research has also been active on their green synthesis [87]. One example was found in the literature that used a bolamphiphile, i.e., HO-Tyr-Leu-Suc-Phe-Tyr-OH, as a hydrogelator for the in situ formation of Pt NPs that were then applied for the hydrogenation in water of p-nitroaniline to p-phenylendiamine [69].

#### 2.1.5. Palladium (Pd)

Pd-based nanostructures have also been widely studied for various applications in catalysis, and the topic has been reviewed in detail [88]. Similarly to the case of platinum, Pd NPs have also been re-discovered in the biomedical field, for applications that are rather diverse as they range from antimicrobial use to cancer therapy and sensing [89]. A similar bolamphiphile to the one mentioned above for platinum NPs, i.e., HO-Try-Trp-Suc-Trp-Tyr-OH was reported as a sonication-induced hydrogelator that templated the in situ formation of Pd NPs which were used as catalysts for the N-deprotection of amino acids and peptides in the presence of sodium borohydride [68].

Keratin is among the cheapest and most widely available natural sources of proteinaceous materials, and is a promising resource to achieve hydrogels [90]. An interesting study used pre-treated human hair to adsorb Pd^2+^ and reduce it to 4 nm-sized NPs through pyrolysis, for the heterogeneous catalytic reduction of nitrobenzene to aniline, which is an industrially-relevant target, being a precursor for a variety of pharmaceuticals, dyes, agricultural products, and polyurethanes [91]. The authors inferred that presence of Cys thiols, as well as other functional groups, in keratin may have promoted NP formation through metal coordination [91]. Therefore we anticipate similar applications also for keratin-derived gels.

#### 2.1.6. Lanthanides

Lanthanide-doped upconverting NPs are able to convert near-infrared excitation into visible and ultraviolet emission. These unique properties find applications in disparate fields that include bioimaging, nanomedicine, and security labeling [92]. A tripodal Phe derivative showed the ability to gel a variety of organic solvents only upon application of ultrasounds; organogels in toluene were successfully used for the in situ formation of tamarium/ytterbium (Tm/Yb) NPs that decorated the surface of the gelling fibers and displayed interesting optical properties that the authors envisaged for the future development of optoelectronic devices [70].

### 2.2. Nanotubes, Nanowires and Nanorods

#### 2.2.1. Gold

**Figure 5:**
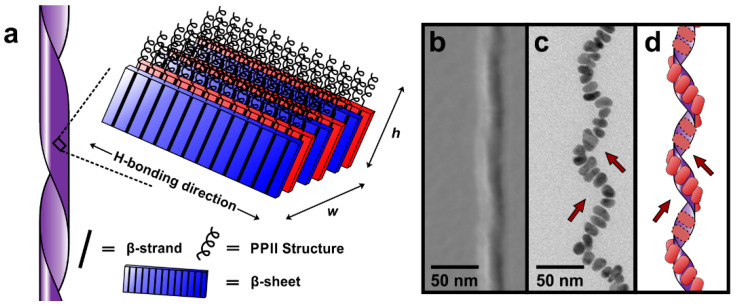
(**a**) Peptide amphiphile assembly model into a nanoribbon. (**b**) AFM image of the resulting assembled fiber. (**c**) TEM image of the gold nanorods oriented (arrows) into a helical pattern. (**d**) Proposed model of the gold nanorods associated to the outer surface of the peptide ribbon. Adapted with permission from [97]. Copyright © 2021, American Chemical Society.

Gold anisotropic nanostructures find important applications that encompass catalysis, sensing, and biomedicine, and their chemical synthesis has been recently reviewed [93]. There is an increasing interest towards the development of differently-shaped gold NPs and how morphology can be used to tune the properties of the final materials [94]. In particular, new methods are continuously sought for the controlled preparation of ultrathin gold nanowires [95], and gold nanorods [96]. A peptide amphiphile gelator was shown to self-assemble into helical nanoribbons and, upon addition of suitable reagents, including a gold salt and a reductant, in situ formation of gold nanorods associated to the outer surface of the nanoribbon was observed, as shown in Figure 5 [97]. Remarkably, the nanorods further associated into a helical superstructure along the ribbon and the authors proposed a mechanism through binding onto the aromatic surfaces of Phe and Tyr side chains, as well as Met functional groups [97].

#### 2.2.2. Silver

Silver nanowires have recently gathered attention for their low cost, high conductivity, transmittance, and availability for applications in flexible electronics [98]. The first example of a supramolecular peptide nanostructure templating their formation goes back to 2003 [63]. Diphenylalanine was then described for its ability to self-assemble into nanotubes, whose hollow nature was exploited for the in situ formation of silver nanowires with the use of a reductant [63]. The peptide could easily be degraded by a hydrolytic enzyme to leave behind the metal nanowires [63]. It was later shown that this dipeptide forms metastable hydrogels [99,100]. Interestingly, while homochiral Phe-Phe is notorious for its uncontrolled hierarchical aggregation into nano- and microtubes, its heterochiral d-Phe-l-Phe isomer self-organizes into 4 nm-wide nanotubes [101] that yield a hydrogel and could offer a way to achieve controlled-size and ultrathin metal nanowires.

#### 2.2.3. Silica

**Figure 6:**
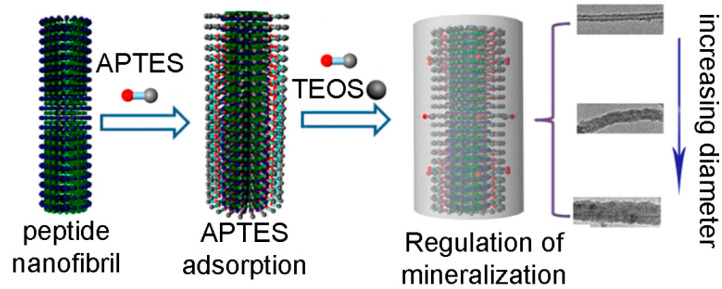
Silica nanotube formation on a peptide nanofibril through the adsorption of APTES and TEOS as precursors. Adapted with permission from [108]. Copyright © 2021, American Chemical Society.

Anisotropic silica nanostructures are promising candidates for advanced applications in the biomedical field, including biocatalysis, biosensing and drug release [102]. In particular, templated-assisted one-dimensional silica nanotubes have been attracting a great deal of interest [102]. We could not find examples in the literature of hybrid or composite gels whereby a peptide was used to template silica nanotubes, however, there are a few examples of peptide nanofibers doing the same in a sol-gel process. In particular, nanotube-forming octapeptide lanreotide was successfully used to template the formation of silica double-walled nanotubes [103]. Cationic peptides are expected to interact well with negatively-charged silicate anions. As an example, Arg-bearing fibrils from an 11 mer peptide templated hollow silica nanotubes more efficiently than an anionic analog [104]. Also Lys can be used to the same end [105], as demonstrated for the fibrillating Ile-Ile-Ile-Lys [106] and Ac-Ile-Ile-Ile-Lys-NH_2_, which, combined with 3-aminopropyltriethoxysilane (APTES) and tetraethyl orthosilicate (TEOS) led to the templating of either bead-strings or uniform nanotubes in a sol-gel process [107]. Interestingly, however, also the anionic peptide Ac-Ile-Ile-Ile-Glu-NH_2_ was successful for the same purpose and adjustment of reaction conditions allowed the control over the hollow silica nanotube diameter from 10 to 60 nm, as shown in Figure 6 [108].

## 3. Conclusions

This mini review highlighted the growing literature on the application of peptide-based gelators to template the in situ formation of metal nanostructures for a variety of applications. This field brings together the tunable properties of gels as well as their attractive feature of controlled embedment and release of guests, with those of inorganic nanoparticles. Not only does this approach provide green and economical ways to prepare innovative nanostructured materials, but also, finds applications in a variety of areas, ranging from biomedicine and sensing to catalysis and environmental detoxification, and so on [109]. Although the field has matured over the years, there is still a lack of understanding of many aspects. There are nonclassical phenomena that are unique to the nanoscale that are worth investigating, for instance with regards to unclear mechanisms of NP nucleation and growth that still need elucidation [110]. Understanding these aspects will provide the basis for a systematic approach towards the development of nanostructured materials for improved performance.

The field of nanomedicine is advancing at rapid pace. NP incorporation into nanogels holds promise in innovative therapy and targeted delivery for reduced side-effects [11,111]. Alternatively, multiple components can be combined together, as recently demonstrated in the case of a PEG hydrogel with gold nanorods, liposomes, and an antimicrobial peptide for the photo-triggered release of the latter [112]. Furthermore, metal coordination can also affect the properties of peptide-based supramolecular hydrogels in ways that are not always straightforward to predict, and further understanding in this area will be important [113]. In conclusion, as we gather further knowledge on the use of supramolecular chemistry based on peptides and green solvents for the templating of metal nanoparticles, we pave the way towards the development of advanced multi-component nanostructured materials for revolutionary applications that will certainly have an impact on society.

## Figures and Tables

**Table 1:** Metal NP formation templated by peptide gelators. n.d. = not determined.

Peptide Gelator	Metal	Solvent	Morphology	Size	Ref.
Ac-Leu-Ile-Val-Ala-Gly-Lys-NH_2_	Ag	Water	Spherical	10–20 nm	[41]
Boc-Tyr-Phe-Tyr-OMe	Ag	Methanol/Water	n.d.	2–10 nm	[60]
Cbz-Val-Val-Val-OMe	Ag	Butanol	Varied	>100 nm	[61]
Cbz-Val-Val-Cys(S-Bzl)-OMe	Ag	Butanol	Spherical	9 nm	[61]
Fmoc-Gly-Cys-Glu-OH	Ag	Water	n.d.	1–2 nm	[46]
Fmoc-Val-Asp-OH	Ag	DMSO/Water	n.d.	1–3 nm	[40]
(*N*-lythocholyl)-Gly-d-Phe	Ag	Water	spherical	5–10, 25 nm	[42]
*N,N,N*-trymethyl-Trp-NH- C_16_H_33_	Ag	Water	Irregular	10–20 nm	[62]
Phe-Phe	Ag	Water	Nanowires	n.d.	[63]
Boc-Phe-Tyr-Phe-OMe	Au	Toluene	n.d.	50–80 nm	[60]
Boc-Tyr-Phe-Tyr-OMe	Au	Toluene	n.d.	15–20 nm	[60]
Boc-Tyr-Phe-Tyr-OMe	Au	Methanol/Water	Spherical/ Hexagonal	15–40 nm	[60]
C_15_H_31_-CO-Val-Trp-OH	Au	Water or Toluene	Spherical	12–14 nm	[64]
C_15_H_31_-CO-Ile-Trp-OH	Au	Water or Toluene	Spherical	12–14 nm	[64]
C_15_H_31_-CO-Val-(*N’*-Me)-Trp-OH	Au	Water or Toluene	Spherical	15–17 nm	[64]
C_15_H_31_-CO-Val-Phe-OH	Au	Water or Toluene	Spherical	15–17 nm	[64]
Fmoc-Phe-Ser	Au	Water	Spherical	7–9 nm	[65]
Fmoc-Phe-Tyr	Au	Water	Rectangular	~140 nm	[65]
*N,N,N*-Trimethyl-Phe-NH-C_16_H_33_	Au	Water	n.d.	5–10 nm	[66]
*N,N,N*-Trimethyl-Phe-NH-C_16_H_33_-NH-(*N*-Boc)-Phe	Au	Water	Octahedral	15–30 nm	[66]
*N,N,N*-Trimethyl-Phe-NH-C_16_H_33_-NH-Phe-(*N*-Boc)-Phe	Au	Water	Octahedral/ Irregular	25–45 nm	[66]
*N,N,N*-Trimethyl-Phe-NH-C_16_H_33_-NH-Ala-(*N*-Boc)-Phe	Au	Water	Octahedral/ Irregular	25–45 nm	[66]
*N,N,N*-Trimethyl-Trp-Pro-NH-C_16_H_33_	Au	Water	2D-Triangular	1 micron	[58]
*N,N*-Dimethyl-Pro-Trp-NH- C_16_H_33_	Au	Water	Nanowires	10 nm	[58]
*N,N,N*-Trimethyl-Trp-Phe-NH-C_16_H_33_	Au	Water	Octahedral	60 nm	[58]
*N,N,N*-Trimethyl-Phe-Trp-NH-C_16_H_33_	Au	Water	Decahedral	210 nm	[58]
Boc-Phe-Val-Phe-OH	Cd	Water	n.d.	6–14 nm	[59]
Boc-Phe-Leu-Phe-OH	Cd	Water	n.d.	6–14 nm	[59]
Boc-Phe-Phe-Phe-OH	Cd	Water	n.d.	6–14 nm	[59]
C_15_H_31_-CO-(Cys)_4_-(Gly)_3_-Sep-Arg-Gly-Asp-OH	Cd	Water	Spherical	3–7 nm	[67]
HO-Tyr-Trp-Suc-Trp-Tyr-OH	Pd	Water	Spherical	3–9 nm	[68]
HO-Tyr-Leu-Suc-Phe-Tyr-OH	Pt	Water	Spherical	1–6 nm	[69]
*N,N’,N”*-tri-((*N*-Boc)-Phe)-1,3,5-benzenetriamine	Tm/Yb	Toluene	Spherical	10–30 nm	[70]
